# The impact of Hyssop (*Hyssopus officinalis*) extract on activation of endosomal toll like receptors and their downstream signaling pathways

**DOI:** 10.1186/s13104-022-06253-3

**Published:** 2022-12-12

**Authors:** Masoumeh Ghasempour, Maryam Hosseini, Mohammad Sadegh Soltani-Zangbar, Roza Motavalli, Leili Aghebati-Maleki, Sanam Dolati, Amir Mehdizadeh, Mehdi Yousefi, Javad Ahmadian Heris

**Affiliations:** 1 Stem Cell Research Center, Tabriz University of Medical Sciences, Tabriz, Iran; 2 Department of Allergy and Clinical Immunology, Pediatric Hospital, Tabriz University of Medical Sciences, Tabriz, Iran; 3 Trauma Research Center, Shahid Rajaee (Emtiaz) Trauma Hospital, Shiraz University of Medical Sciences, Shiraz, Iran; 4 Student Research Committee, Tabriz University of Medical Sciences, Tabriz, Iran; 5 Department of Immunology, School of Medicine, Tabriz University of Medical Sciences, Tabriz, Iran; 6 Molecular Medicine Research Center, Tabriz University of Medical Sciences, Tabriz, Iran; 7 Immunology Research Center, Tabriz University of Medical Sciences, Tabriz, Iran; 8 Physical Medicine and Rehabilitation Research Center, Aging Research Institute, Tabriz University of Medical Sciences, Tabriz, Iran; 9 Hematology and Oncology Research Center, Tabriz University of Medical Sciences, Tabriz, Iran

**Keywords:** Hyssop, TLR, Immune system, Proinflammatory cytokines, IFN-I

## Abstract

**Objectives:**

From the ancient, medicinal benefits of Hyssop (*Hyssopus officinalis L.*) have been implicated for respiratory and digestive diseases despite the effects of Hyssop on viral infections have not been mechanistically investigated. In this study, we examined whether the Hyssop extract activated anti-viral innate immunity, as a sentinel for immune system, through activation of endosomal TLRs recognizing nucleic acids and their downstream signaling. The Hyssop herb extracts was prepared and co-cultured with healthy individual’s peripheral blood mononuclear cells (PBMCs). After viability assay, gene expression levels of TLR3,7,8,9, as well as MyD88 and NF-κB, were evaluated in treated PBMCs using Real-time PCR. Next, the secretion level of immune related cytokines was quantified via ELISA.

**Results:**

Post 24 h, 40 µg/ml of the extract significantly inhibited the viability of less than 50% of cells compared to the control and had a maximum effect on cellular function. The Hyssop-treated PBMCs demonstrated an elevated expression of endosomal TLRs genes, as well as MyD88 and NF-κB. Moreover, the release of INF-α and β notably enhanced in cell culture supernatant, while the content of inflammatory cytokines remarkably diminished (P < 0.05). The Hyssop extract was capable of inducing antiviral innate immune responses so can be promising in antiviral drug strategies.

**Supplementary Information:**

The online version contains supplementary material available at 10.1186/s13104-022-06253-3.

## Introduction

One of the most important plants in the traditional medicine is Hyssop (*Hyssopus Officinalis L*.), belonging to Lamiaceae family, used in flavored beverages from the ancient. In Iran, Hyssop plant is grown spontaneously in the northwestern and southeastern of Iran, and the regions around the Caspian Sea [1]. According to previous studies, the Hyssop plant possesses excessive medicinal properties such as antifungal, antibacterial and antiviral activities, which have made it a beneficial folk medicine to alleviate digestive and intestinal disorders, as well as respiratory diseases such as tuberculosis, asthma, chronic catarrh, and bronchitis [2, 3]. Srivastava et al. have reported the effectiveness of Hyssop in the treatment of nose, throat, and lung afflictions because of its anti-inflammatory properties in Southern Europe [4]. Nevertheless, these therapeutic applications and health benefits of Hyssop have been documented mainly based on traditional medicine observations rather than scientific evidences [5]. Indeed, there are not enough scientific data regarding the mechanism of immune system activation via Hyssop herb.

With the recent COVID-19 outbreak, a discussion has emerged about the potential values of traditional medicine in the prevention and treatment of the disease when modern medicine appears to be disabled to overcome it. Viral illnesses, with their fascinating dynamics of causative organisms are undoubtedly at the top of our wish list [6]. It has been believed that innate immune system acts as the first responder for the detection and clearance of infections. Innate immune cells secrete various cytokines which inhibit viral replication, stimulate the adaptive immune response, and recruit other immune cells to the site of infection [7–10]. Considerably, Toll-like receptors (TLRs) are responsible for sensing invading microbes in different parts of a cell, such as the plasma membrane (TLR1, TLR2, TLR4, TLR5, TLR6, and TLR11) or in intracellular endosomes (TLR3, TLR7, TLR8, TLR9, and TLR10) [11]. TLRs trigger innate immune responses through activation of signaling cascades depending on the adaptors myeloid differentiation primary response protein 88 (MyD88) or TIR-domain-containing adapter-inducing interferon-β (TRIF), and then sequentially induce the production of pro-inflammatory cytokines, type I interferons (IFNs-I), chemokines, and antimicrobial proteins through transcription factors activation such as nuclear factor kappa B (NF-κB) [12].

According to these evidences, this study aimed to investigate the impact of Hyssop extract on innate immune response through activation of TLRs and probable signaling pathways in anti-viral responses.

## Main text

### Materials and methods

#### PBMCs isolation from blood samples

PBMCs were isolated from heparinized blood samples of 45 healthy individuals (for all assays) and 20 COVID-19 patients (for MTT assay only) by Histopaq density gradient centrifugation (Sigma, Missouri, USA) after receiving informed consent from the participants. In details, the obtained samples were diluted with the same volume of PBS. Then, the Ficoll-Paque Plus (Biosra, France) was carefully added to diluted samples in 1:2 ratio respectively. The mixture was then centrifuged at 400 × g for 20 min at 20 °C. The undisturbed PBMC layer was carefully transferred to a new tube and washed twice with three volumes of PBS. This study was approved by the Ethics Committee in Tabriz University of Medical Sciences (Code: IR.TBZMED.REC.1399.1143).

#### Extraction of plant extracts and treatment with PBMCs

Plant extracts was extracted from 10 g of crushed *h*yssopus samples using ultrasonic bath at 20 kHz and 100 ml of ethanol (80%) at 45 °C for 20 min. Then, the solvents was evaporated by vacuum evaporator SPS:refid::bib13(13). The Hyssop extract was then treated with PBMCs (1 × 10^6^ cells/well) in complete RPMI-1640 medium (Gibco, Paisley, UK) supplemented 15% heat-inactivated fetal bovin serum (FBS; Gibco, Paisley, UK), 100 mg/ml streptomycin, 100 u/ml penicillin (Gibco) and 2 mM L-glutamine (Gibco, Paisley, UK) for 12, 24 and 48 h at 37 °C in 5% atmospheric CO2 and 95% humidity. Simultaneously, PBMCs were stimulated with suboptimal dose (10 ng/ml) of phorbol myristate acetate (PMA; Gibco, NY, USA).

#### Cell viability

The viability of PBMCs was measured by the methyl thiazol tetrazolium bromide (MTT) assay. Briefly, 2 × 10^5^ cells/well were seeded in a 96-well plate. The cells were treated with increasing concentrations of Hyssop extract (5–50 µg/ml) for 24 h. Subsequently, 100 ul of MTT (1 mg/ml, Sigma, USA) was added into each well and incubated for 4 h at 37 °C. Then, 100 µl DMSO was added to each well to dissolve the purple formazan crystals and incubated in room temperature for 30 min. Later, the optical density was measured using a spectrophotometer at 540 nm wavelength and compared to untreated cells [14].

#### Gene expression assay

The expression levels of genes including TLR 3,7,8,9, as well as NF-κB and Myd88 genes were assessed in PBMCs treated with Hyssop extract by real-time polymerase chain reaction (PCR). Briefly, total RNA was extracted from cultured PBMCs using RNA extraction kit (Qiagen, Hamburg, Germany) according to the manufacturer’s instructions. Then, cDNA synthesis was synthesized by the usage of a cDNA synthesis kit (Exiqon, Copenhagen, Denmark), based on manufacturer’s instructions. The expression level of each mRNA was measured by real-time PCR and a SYBR Green Real Time PCR kit (Ampliqon), according to manufacturer’s instructions. Finally, the mRNA expression was normalized by the detection of β-actin housekeeping gene. The used primers sequences designed by OLIGO v. 7.56 software (Molecular Biology Insights, Inc., CA, USA) for qPCR assay are listed in Additional file 1: Table S1.

##### Quantification of cytokine levels

The concentrations of tumor necrosis factor (TNF)-α, interleukin (IL)-1β, IL-8 and IFN I α and β cytokines were quantified using ELISA kits (Mybiosource, San Diego, USA), according to the manufacturer’s instruction. Briefly, the wells of micro-titer ELISA plate (Maxisorp, Nunc, Roskilde, Denmark) were coated with anti-TNF-α, anti-IL-1β, anti-IL-8 and anti-IFN I α and β antibodies. Afterward, the supernatant of the cells and biotinylated mouse anti-hTNF-α mAb, anti-hIL-1β mAb, anti-hIL-8 mAb and anti-hIFN I α and β mAb were added to the relative wells. Next, the existence of anti-cytokine antibody was investigated by the addition of a streptavidin alkaline phosphatase conjugated anti-mouse IgG Ab (Sigma). Finally, p-nitrophenyl phosphate (4 mg/ml) was added as substrate and the absorbance was evaluated at 405 nm in an ELISA plate reader (LabSystems, Helsinki, Finland).

#### Statistical analysis

All experiments were performed in triplicate. Achieved data were analyzed using GraphPad Prism v.8 (GraphPad, La Jolla, CA) and presented as mean ± standard deviation (SD). *One-Way ANOVA followed by Dunnett's T3 multiple comparisons test* was done to compare the means between groups. *p* < 0.05 was considered as statistically significant.

## Results

### Hyssop extract decreased the PBMCs cells viability

To determine the cytotoxic effect of Hyssop extract on PBMCs, the cells were treated with multiple concentrations of the extract. Our results depicted the proliferation rate of cells was inhibited as the extract concentration enhanced during 24 h, so that the IC50 value of the Hyssop extract was about 45 µg/ml (Additional file 2: Fig. S1A). Almost similar results were observed on PBMC of COVID-19 patients (Additional file 2: Fig. S1B). According to this, a sub-cytotoxic concentration of 40 µg/ml herbal extract, lower than its IC50 value, was used for the forward examinations.

### Hyssop extract increased the TLRs and down-stream molecules expression level

**Fig. 1:**
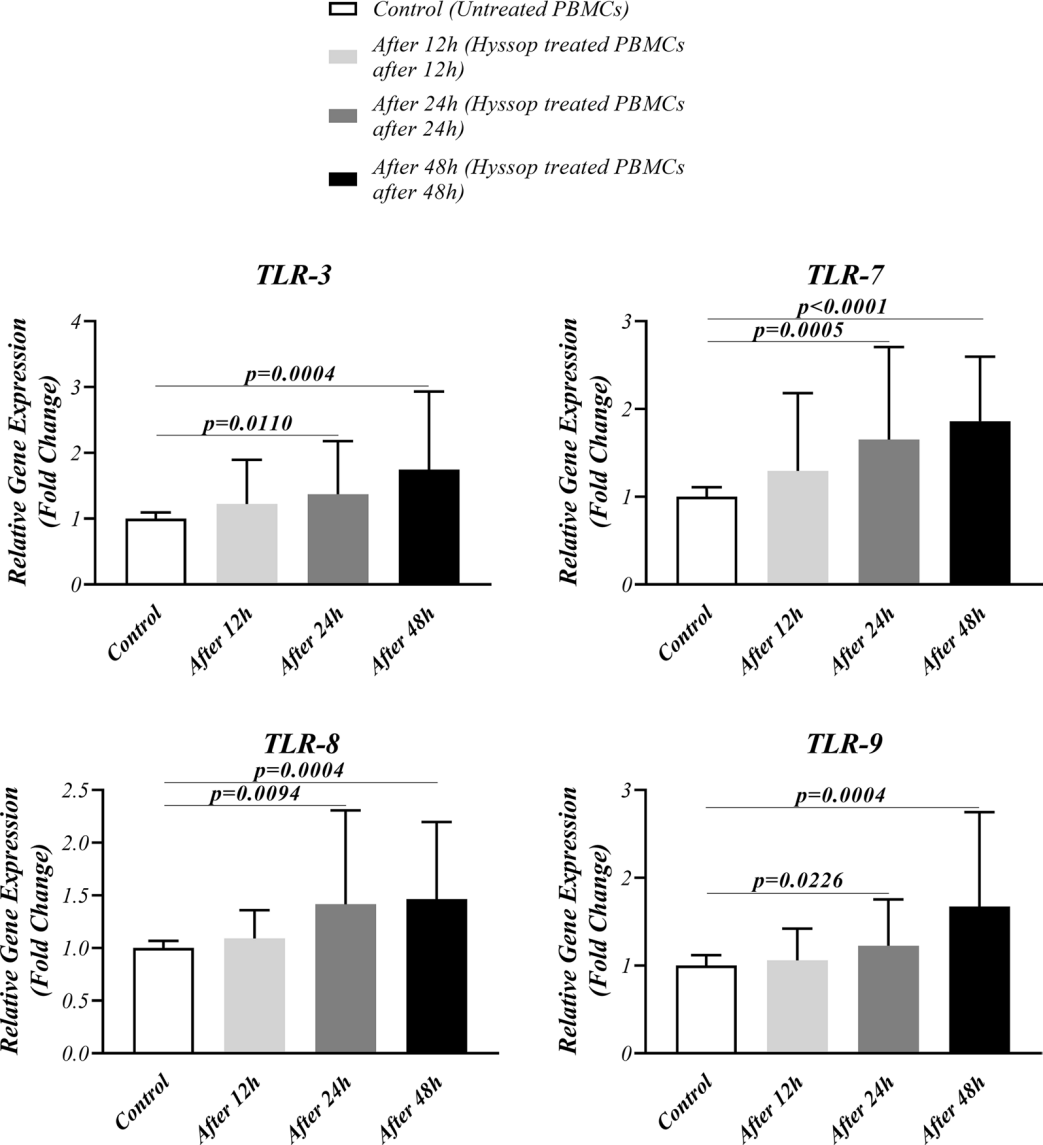
Gene expression levels of endosomal TLRs. The fold changes of TLR 3, 7, 8, and 9 gene expressions were estimated in PBMCs treated with 40 µg/ml Hyssop extract in comparison to the control following 12, 24 and 48 h using real time PCR. The experiment was performed in triplicate. Data are presented as mean ± standard division (SD). P < 0.05 was considered as statistically significant

The expression levels of TLR 3, 7, 8, 9 genes, as well as Myd88 and NF-κB genes were evaluated in Hyssop extract treated cells using real-time PCR. As illustrated in Fig. 1, after 24 and 48 h, the fold changes of TLR 3, 7, 8, and 9 genes were remarkably increased in the treated cells when compared with the control group. Nonetheless, the differences of the expression levels of each TLR genes during 24 and 48 h were not statically significant. Moreover, Myd88 expression level was notably increased in the treated cells after 24 h and 48 h. However, NF-κB expression was significantly increased after 48 h, only compared to control group (Additional file 3: Fig. S2, Table 1).

**Table 1:** Gene and protein expression of TLRs and downstream molecules in studied population

Target	Control (mean ± SD) (N = 45)	After 12 h (mean ± SD) (N = 45)	After 24 h (mean ± SD) (N = 45)	After 48 h (mean ± SD) (N = 45)	p value		
					Control vs. After 12 h	Control vs. After 24 h	Control vs. After 48 h
Relative gene expression (Fold change)							
*Myd88*	1.001 ± 0.08261	1.188 ± 0.5632	1.599 ± 0.4567	1.667 ± 0.6941	NS	< 0.0001	< 0.0001
*NFқB*	1.000 ± 0.07271	1.060 ± 0.5233	1.218 ± 0.6694	1.395 ± 0.4382	NS	NS	< 0.0001
*TLR-3*	1.000 ± 0.09613	1.220 ± 0.6738	1.371 ± 0.8069	1.746 ± 1.184	NS	0.0110	0.0004
*TLR-7*	1.000 ± 0.1076	1.294 ± 0.8883	1.652 ± 1.055	1.860 ± 0.7372	NS	0.0005	< 0.0001
*TLR-8*	1.000 ± 0.06735	1.090 ± 0.2698	1.416 ± 0.8921	1.463 ± 0.7341	NS	0.0094	0.0004
*TLR-9*	1.000 ± 0.1192	1.059 ± 0.3625	1.226 ± 0.5289	1.673 ± 1.075	NS	0.0226	0.0004
Enzyme-linked immunosorbent assay (pg/ml)							
IFN-Iα	89.56 ± 34.96	102.2 ± 47.01	120.1 ± 52.12	140.1 ± 57.64	NS	0.0048	< 0.0001
IFN-Iβ	95.31 ± 30.82	111.4 ± 38.57	132.7 ± 49.70	147.9 ± 52.39	NS	0.0002	< 0.0001
IL-1β	64.91 ± 33.20	56.36 ± 28.35	47.02 ± 26.06	40.07 ± 24.50	NS	0.0167	0.0004
TNF-α	36.87 ± 19.50	30.44 ± 15.55	25.84 ± 12.09	22.71 ± 10.46	NS	0.0057	0.0002
IL-8	121.8 ± 44.87	107.8 ± 43.71	94.51 ± 42.57	89.56 ± 38.08	NS	0.0118	0.0012

*MyD88* Myeloid differentiation primary response 88, *NFκB* Nuclear factor kappa B, *TLRs* Toll-Like receptors, *IFN-Is* Type I interferons; ILs: Interleukins, *TNF-α* Tumor necrosis factor alpha. Data are presented as mean ± standard division (SD). P < 0.05 was considered as statistically significant

### The influence of Hyssop extract on cytokine secretions from PBMCs

**Fig. 2:**
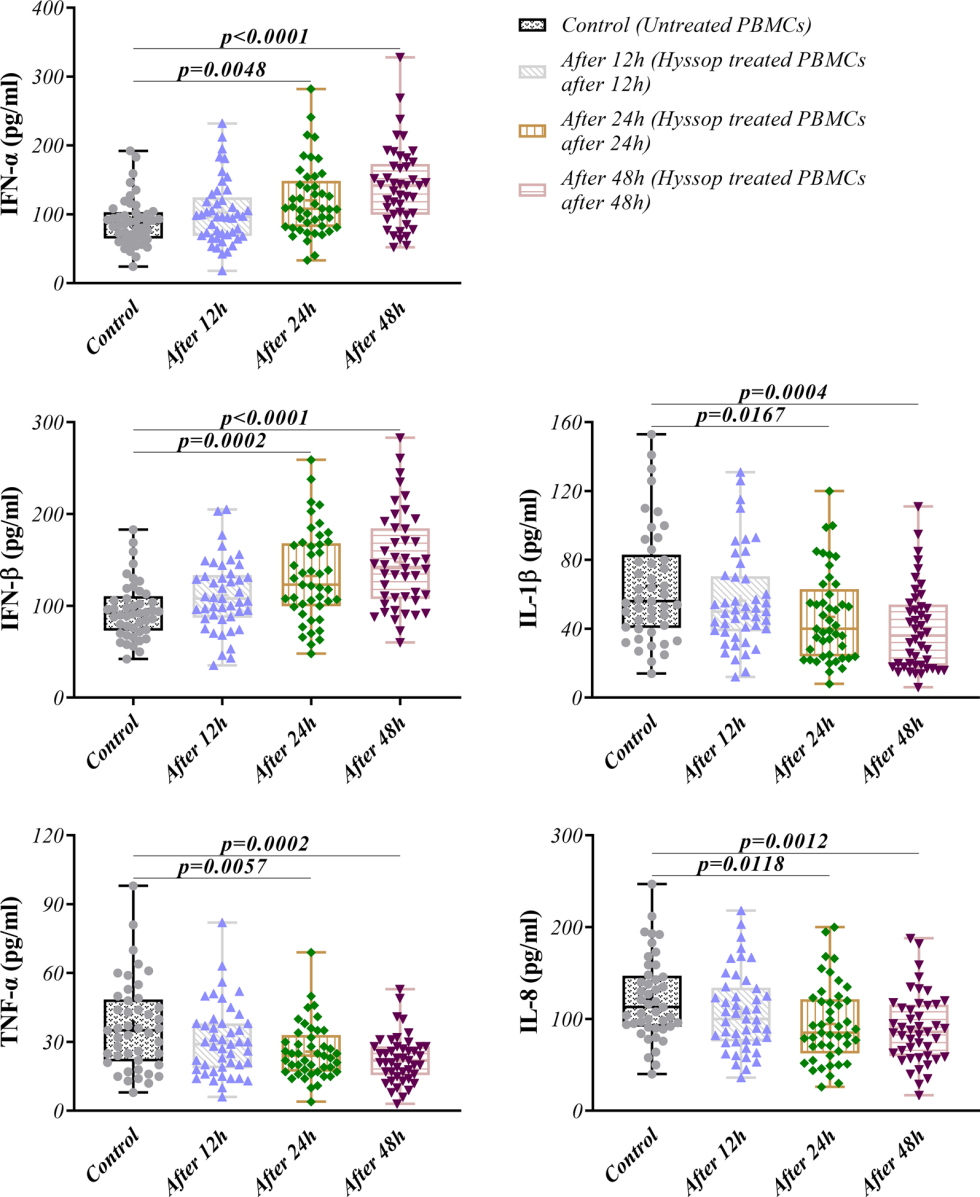
Protein expression levels of cytokines. The expression levels of IFN-α and β, IL-1β, TNF-α and IL-8 proteins were quantitatively assessed by ELISA test in PBMCs treated with 40 µg/ml Hyssop extract in comparison to the control after 12, 24 and 48 h. The experiment was performed in triplicate. Data are presented as mean ± standard division (SD). P < 0.05 was considered as statistically significant

The levels of TNF-α, IL-1β, IL-8 and IFN I α and β cytokines secreted from the cells treated with Hyssop extract were determined using ELISA. The results showed that Hyssop extract had a significant increasing effect on IFN-Iα and IFN-Iβ production in treated cells after 24 and 48 h compared to the control group. However, at the same time points, the concentration of TNF-α, IL-1β and IL-8 were remarkably decreased in supernatants of treated cells in comparison to the untreated control (Fig. 2, Table 1).

## Discussions

In this study, the role of Hyssop plant in induction of anti-viral innate immune response was assessed. Our results exhibited that the Hyssop extract can significantly enhance the expression levels of endosomal TLRs genes including TLR 3,7,8,9 and their downstream signaling pathway molecules.

In folk medicine, Hyssop has been used as an anti-inflammatory, anti-catarrhal, and antispasmodic drug in different nations [4, 5]. In parallel, from the scientific point of view, it has been declared that *Hyssopus officinalis* effectively regulates the secretion of IL-4, IL-17 and interferon-γ (IFN-γ), as well as T helper (Th) 1/Th2 cytokines imbalance in asthmatic mouse model [15–17]. However, it is unclear how Hyssop extract influences innate immune responses, which undoubtedly acts a critical role in restriction of infection and inhibition of pathogen invasion [18]. Thus, we investigated the impact of this herb extract on TLR signaling pathways, as the main pattern recognition receptors (PRRs) in innate immune responses [19]. On the other hand, it has been highlighted that tea blends of Hyssop herb are effective in cough relief, and treatment of laryngitis [4]. According to this, we assumed that Hyssop plant is beneficial for viral infections such as COVID-19 which affect respiratory tracts. Our results indicated that the fold changes of endosomal TLRs including TLR 3,7,8,9 gene expressions significantly enhanced in PBMCs treated with Hyssop extract following 24 and 48 h. As such, the gene expression level of Myd88 and NF-κB notably elevated in these cells in comparison to the control. It is well accepted that endosomal TLRs recognize microbial nucleic acids especially viral nucleic acids [20]. TLR 7, 8,and 9 employ Myd88 molecule to direct their signaling pathway; while, TLR3 activation is transferred through the TRIF molecule [21]. Finally, MyD88/TRIF-NF-κB signaling cascade induces the production of cytokines such as TNF-α, IL-6, IL-1 and IFN-I [21]. Based on coronavirus-related studies findings, TLR 3&7 are the most likely candidates recognizing pathogen-associated molecular patterns, which are considered as important immune mechanisms controlling infection, particularly SARS-CoV-2 [22]. Sallenave et al. demonstrated that TLR 2, 3, 7 and 8 can stimulate the production of antiviral pro-inflammatory mediators (IL-6, IL-8, IFNs) in epithelial and myeloid cells following the activation of NF-κB [23]. However, our results displayed that the secretion of IFN-α and β are significantly increased in supernatants of the treated cells, while the inflammatory cytokines including IL-8, IL-1β and TNF-α are considerably diminished in these cells. This may raise the hypothesis that the inhibitory effect of Hyssop on inflammation is independent from endosomal TLRs signaling; whereas, the herb extract stimulates IFN-I release through endosomal TLRs activation. Previously, Liu et al. demonstrated that hyssopuside (HY), a novel phenolic glycoside isolated from *Hyssopus cuspidatus*, were able to reduce nitric oxide (NO) production and hamper the production of pro-inflammatory mediators in LPS-stimulated macrophages [24]. Meanwhile, whether TLRs are involved in the inhibition of poinflammatory processes via Hyssop needs further researches.

Blanco-Melo et al. also reported that IFN-I can efficiently restrict SARS-CoV-2 replication in vitro, suggesting that SARS-CoV-2 triggers an IFN-I response which could limit viral spread [25]. Furthermore, a clinical study reported that IFN-I were not detected (particularly IFN-β) or at least at lower levels (IFN-α) in plasma of patients with severe COVID-19 [26, 27]. Some studies have also described a direct relationship between the severity of COVID-19 and over-production of proinflammatory cytokines [9, 28–30].

## Conclusion

We showed that the Hyssop extract can significantly induce antiviral cytokine (INFs-I) production in PBMCs possibly through endosomal TLRs and their downstream signaling pathways. Moreover, the Hyssop has the capability to impede the release of proinflammatory cytokines from the PBMCs suggesting the potential usage of Hyssop in antiviral drugs. Although, it needs ongoing examinations on mechanisms by which the plant exerts anti-inflammatory consequences.

## Limitations

A limitation of our study is the lack of functional assay.

## Supplementary Information

**Additional file 1: Table S1.** Primer sequences of the evaluated genes

**Additional file 2: Fig S1. **Cell viability.** A** Healthy individuals’ peripheral blood mononuclear cells (PBMCs) were treated with increasing concentrations of the Hyssop extract for 24 h and the viability of the cells were evaluated using MTT assay in comparison to untreated cells. **B** COVID-19 patients’ peripheral blood mononuclear cells (PBMCs) were treated with increasing concentrations of the Hyssop extract for 24 h and the viability of the cells were evaluated using MTT assay in comparison to untreated cells. The experiment was performed in triplicate. Data are presented as mean ± standard division (SD). P < 0.05 was considered as statistically significant. *Represents for P < 0.05.

**Additional file 3: Fig S2. **Gene expression levels of Myd88 and NF-κB. The fold changes of Myd88 and NF-κB gene expressions were assessed in PBMCs treated with 40 µg/ml Hyssop extract in comparison to the control after 12, 24 and 48 h using real time PCR. The experiment was performed in triplicate. Data are presented as mean ± standard division (SD). P < 0.05 was considered as statistically significant.

## Data Availability

The data cannot be shared in public because of ethics and individual privacy restrictions but are limitedly available by contacting the corresponding author of this study, privately.

## Abbreviations

MyD88: Myeloid differentiation primary response protein 88
TLRs: Toll like receptors
NF-κB: Nuclear factor kappa B
IFNs-I: Type I interferons
TRIF: TIR-domain-containing adapter-inducing interferon-β
PBMCs: Peripheral blood mononuclear cells
MTT: Methyl thiazol tetrazolium
PCR: Polymerase chain reaction
TNF-α: Tumor necrosis factor alpha
IL: Interleukin
