# Supplementary material for: The impact of Hyssop (Hyssopus officinalis) extract on activation of endosomal toll like receptors and their downstream signaling pathways
Source: BMC Res Notes. 2022 Dec 12;15:366. doi: 10.1186/s13104-022-06253-3 (PMC9742021; doi:10.1186/s13104-022-06253-3)
Supplement: Supplementary file 1 — Additional file 1: Table S1. Primer sequences of the evaluated genes [file 13104_2022_6253_MOESM1_ESM.docx]

**Additional file 1: Table S1.**  Primer sequences of the evaluated genes

| **Gene** | **Primer** | **Sequence (5′→3′)** |
| --- | --- | --- |
| ***Myd88*** | Forward  Reverse | GAGGCTGAGAAGCCTTTACAGG  GCAGATGAAGGCATCGAAACGC |
| ***NFқB*** | Forward  Reverse | GCAGCACTACTTCTTGACCACC  TCTGCTCCTGAGCATTGACGTC |
| ***TLR-3*** | Forward  Reverse | GCGCTAAAAAGTGAAGAACTGGAT  GCTGGACATTGTTCAGAAAGAGG |
| ***TLR-7*** | Forward  Reverse | CTTTGGACCTCAGCCACAACCA  CGCAACTGGAAGGCATCTTGTAG |
| ***TLR-8*** | Forward  Reverse | ACTCCAGCAGTTTCCTCGTCTC  AAAGCCAGAGGGTAGGTGGGAA |
| ***TLR-9*** | Forward  Reverse | TGAGCCACAACTGCATCTCGCA  CAGTCGTGGTAGCTCCGTGAAT |
| **β-actin** | Forward  Reverse | CACCATTGGCAATGAGCGGTTC  AGGTCTTTGCGGATGTCCACGT |

Abbreviations: MyD88: Myeloid differentiation primary response 88; NFκB: Nuclear factor kappa B; TLRs: Toll-Like receptors.
