# Supplementary figures and images for: The impact of Hyssop (Hyssopus officinalis) extract on activation of endosomal toll like receptors and their downstream signaling pathways
Source: BMC Res Notes. 2022 Dec 12;15:366. doi: 10.1186/s13104-022-06253-3 (PMC9742021; doi:10.1186/s13104-022-06253-3)

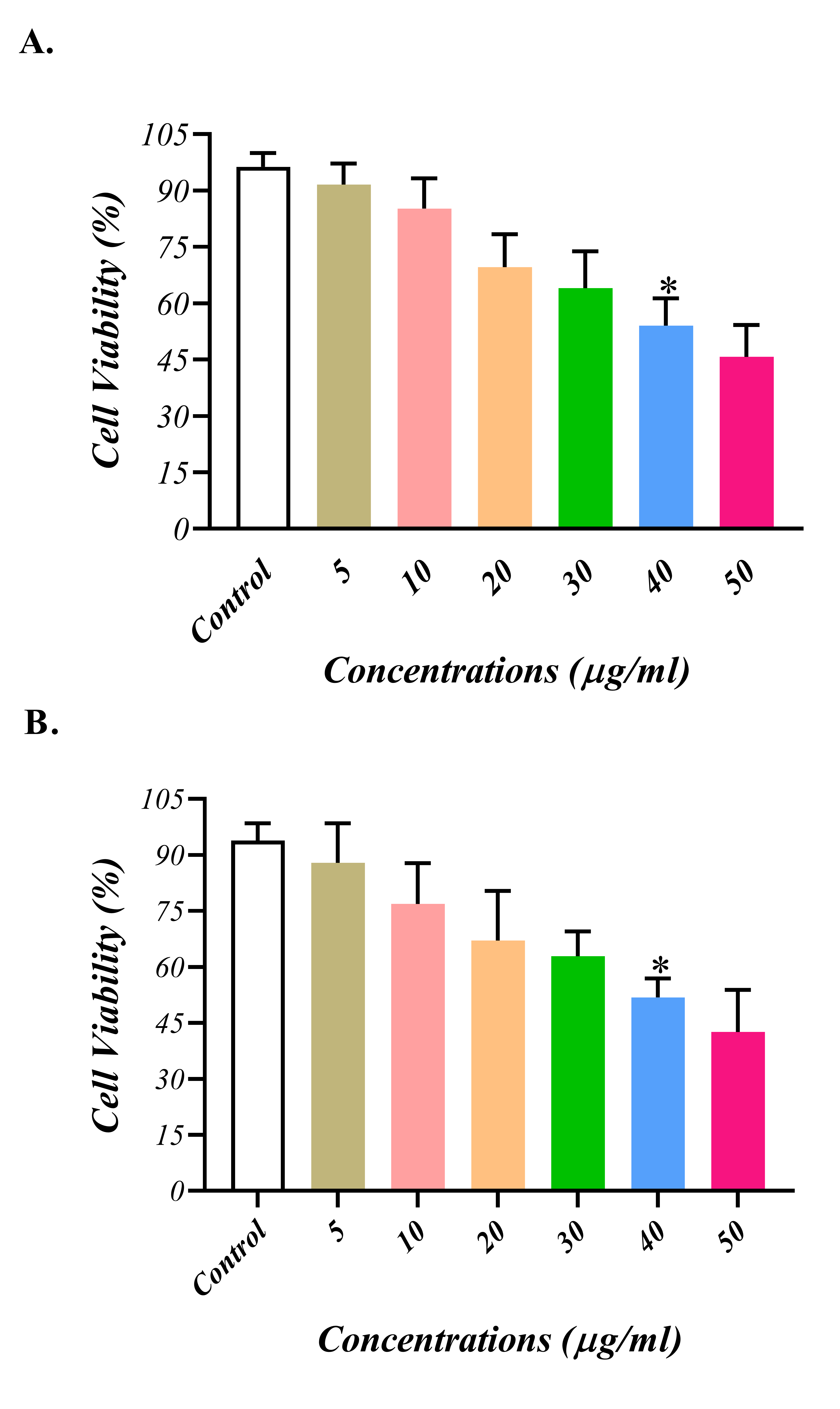

Supplement: Supplementary file 2 — Additional file 2: Fig S1. Cell viability. A Healthy individuals’ peripheral blood mononuclear cells (PBMCs) were treated with increasing concentrations of the Hyssop extract for 24 h and the viability of the cells were evaluated using MTT assay in comparison to untreated cells. B COVID-19 patients’ peripheral blood mononuclear cells (PBMCs) were treated with increasing concentrations of the Hyssop extract for 24 h and the viability of the cells were evaluated using MTT assay in comparison to untreated cells. The experiment was performed in triplicate. Data are presented as mean ± standard division (SD). P < 0.05 was considered as statistically significant. *Represents for P < 0.05. [file 13104_2022_6253_MOESM2_ESM.tif]

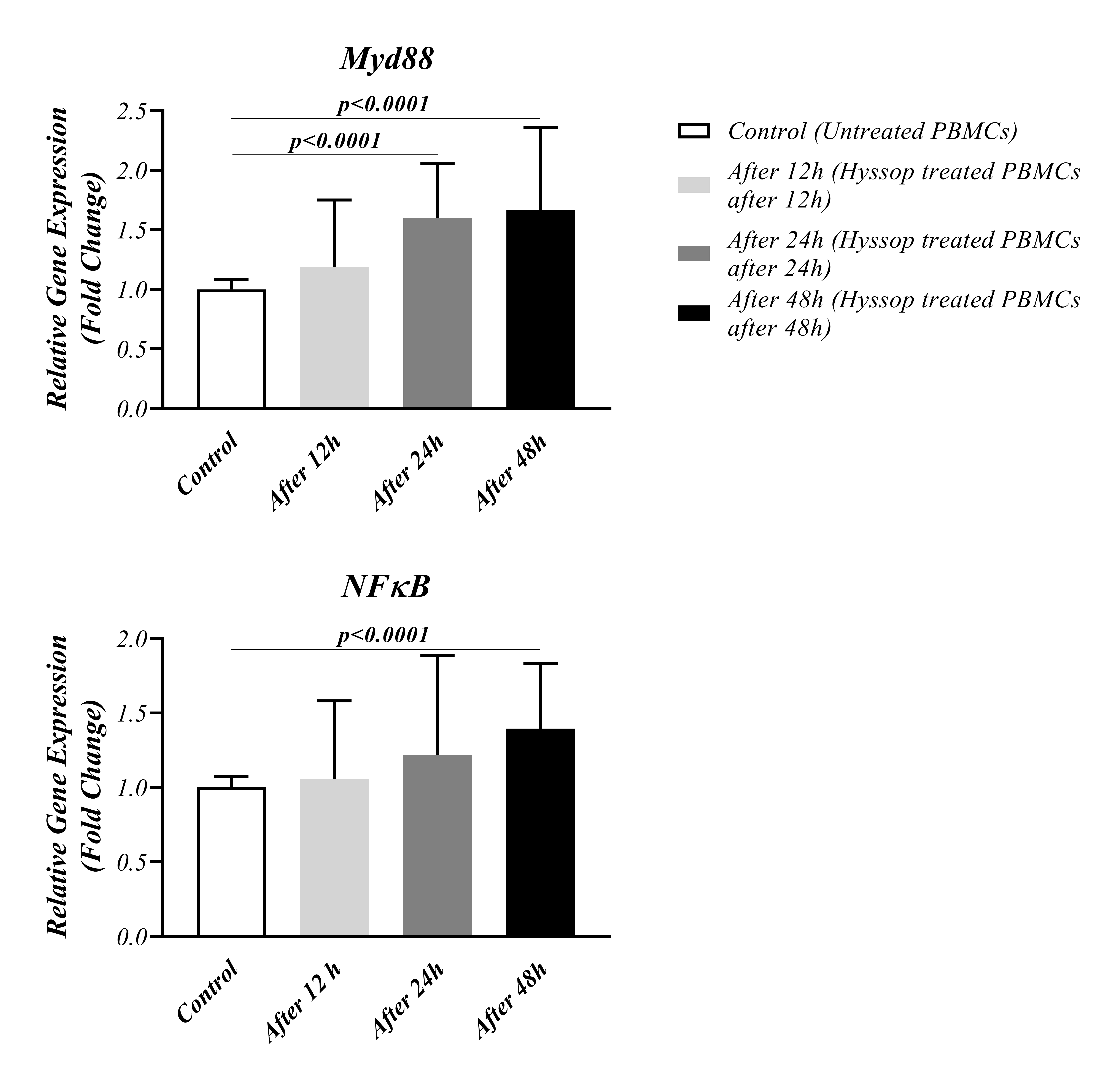

Supplement: Supplementary file 3 — Additional file 3: Fig S2. Gene expression levels of Myd88 and NF-κB. The fold changes of Myd88 and NF-κB gene expressions were assessed in PBMCs treated with 40 µg/ml Hyssop extract in comparison to the control after 12, 24 and 48 h using real time PCR. The experiment was performed in triplicate. Data are presented as mean ± standard division (SD). P < 0.05 was considered as statistically significant. [file 13104_2022_6253_MOESM3_ESM.tif]
